# Antithrombotic properties of JJ1, a potent and novel thrombin inhibitor

**DOI:** 10.1038/s41598-017-13868-1

**Published:** 2017-11-01

**Authors:** Wonhwa Lee, Suyeon Lee, Joonhyeok Choi, Jun-Hyeong Park, Kyung-Min Kim, Jun-Goo Jee, Jong-Sup Bae

**Affiliations:** 1 College of Pharmacy, CMRI, Research Institute of Pharmaceutical Sciences, BK21 Plus KNU Multi-Omics based Creative Drug Research Team, Kyungpook National University, Daegu, 41566 Republic of Korea; 2 Division of Plant Biosciences, School of Applied BioSciences, College of Agriculture and Life Science, Kyungpook National University, Daegu, 41566 Republic of Korea

## Abstract

The development of new anticoagulants is an important goal for the improvement of thrombosis treatment. Recent studies have suggested the importance of thrombin inhibitors in the modulation of thromboembolic disorders. The aim of this study was to discover a new small-molecule thrombin inhibitor. In this study, the compound **JJ1**, which has a novel scaffold, was selected by structure-based docking simulation to determine its potential inhibitory activity against thrombin. **JJ1** was shown to inhibit the catalytic activity of human α-thrombin with a *K*_*i*_ of 0.019 μM by direct binding to the active site and with at least 10,000-fold selectivity relative to that reported for the inhibition of other biologically important serine proteases. **JJ1** prolonged clotting times (activated partial thromboplastin time and prothrombin time) and inhibited the activity and production of thrombin. Furthermore, it inhibited thrombin-catalyzed fibrin polymerization and platelet aggregation. Similar to its *in vitro* antithrombotic activities, **JJ1** showed enhanced antithrombotic effects in an *in vivo* pulmonary embolism and arterial thrombosis model. It also exhibited anticoagulant effects in mice. Collectively, these results demonstrated that **JJ1** was a potent, direct, and selective thrombin inhibitor that may be useful in the management of various thrombotic disorders.

## Introduction

Thrombin is an important multifunctional serine protease that is central to the bioregulation of hemostasis and thrombosis^1^. Thrombin cleaves fibrinogen to form insoluble fibrin and acts as a powerful agonist for the activation and aggregation of platelets, which are critical to the formation of venous and arterial thrombosis, respectively^2^. Normally, thrombin generation is stringently controlled; however, under pathological conditions, excessive thrombin generation occurs. As active thrombin remains in the thrombus after clot formation, it exhibits its activity in the form of direct stimulatory action on endothelial and smooth muscle cell proliferation, as well as on the synthesis and release of prostacyclin, platelet-activating factor, and platelet-derived growth factor^3,4^. As a result, inhibition of thrombin activity and thrombin generation has become an attractive therapeutic target.

Thromboembolic disorders, such as myocardial infarction, stroke, and deep vein thrombosis, continue to be a major cause of morbidity and mortality in the western world^5^. Arterial thrombosis contributes to unstable angina and peripheral arterial occlusion and may lead to acute myocardial infarction or thrombotic stroke^6^. The past decade has seen major progress in the development of antithrombotic agents that are tailored to exhibit antiplatelet activity, aid in the lysis of blood clots, or affect the activity and generation of thrombin. Although heparin and other vitamin K antagonists such as coumarin derivatives are essential components of anti-thrombotic treatment, both drugs have well-known side effects such as a narrow therapeutic window and a highly variable dose-response relationship. These limitations drive the continual and intense effort to develop new anticoagulants, which predominantly target specific coagulation factors^7^. Direct thrombin inhibitors (DTIs) represent potentially useful drugs for the treatment of both venous and arterial thrombosis^8–11^. They are small, synthetic, and specific inhibitors of thrombin that are independent of antithrombin action^12^. Importantly, they can penetrate the thrombus to inhibit fibrin-bound thrombin^13^.

The development of a new drug is a complex process that requires time and money. Computational aids have contributed to the facilitation of early drug discovery processes. Virtual screening, which is used to identify bioactive small molecules, has been developed through the improvement of algorithms and computational capacity. After a reduction in the number of candidate molecules to several tens or hundreds of compounds, researchers have confirmed the activity of each toward target biomolecules using empirical experiments. Two methods, ligand-based and structure-based, are important in virtual screening; however, structure-based virtual screening (SBVS) is more suited for finding novel scaffolds^14,15^. In this study, we utilized SBVS to find novel DTIs. In this study, we selected the optimal structure from among the x-ray structures to improve the efficiency of SBVS by performing test runs with the known ligands and their physicochemically matched decoys, before conducting high-throughput SBVS^16^.

Of the many potential inhibitor molecules, **JJ1** showed antithrombotic activities in cells and *in vivo*. The present report describes the kinetic properties and antithrombotic efficacy of this novel thrombin inhibitor via evaluation of *in vitro* and *in vivo* clotting times, fibrin polymerization, platelet aggregation, fibrinopeptide A (FPA) formation, thrombus formation, and thrombin activity and production.

## Results

### High-throughput structure-based virtual screening

**Figure 1:**
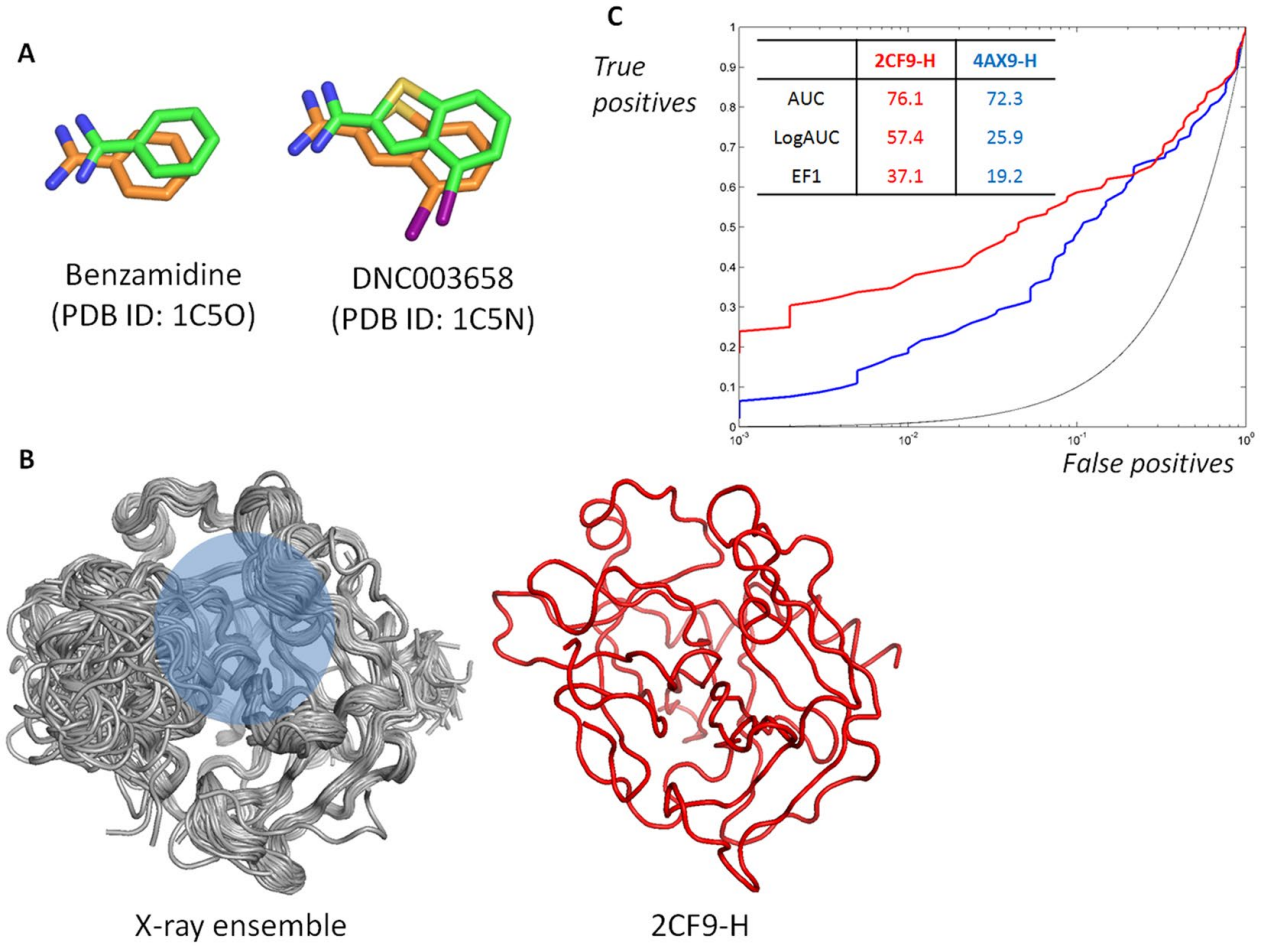
Selection of software and structure for high throughput structure-based virtual screening (SBVS). (**A**) Reproducibility of inhibitor poses observed in x-ray structures by docking with DOCK 3.6. Two molecules, benzamidine and DNC003658 (4-iodobenzothiophene-2-carboxamidine), extracted from the complex structures of 1C5O and 1C5N, respectively, are shown in green, whereas docked structures are shown in orange. The PDB IDs that contain the molecules are labeled in parentheses. (**B**) X-ray ensemble and the selected structure of human thrombin for the current study. The sites for inhibitor binding are shaded and the corresponding PDB ID is shown. (**C**) Receiver operating characteristic (ROC) curves from the selected structures. The profiles from the structure for SBSV (red) and the one that shows the closest values to the averaged values (blue) are drawn for comparison. The percentage values of three metrics, area under the curve (AUC), logarithmically scaled AUC (LogAUC), and enrichment factor at 1% (EF1) of the ROC curve, are also shown. For emphasizing the early enrichments, the x-axis was scaled with Log_10_(x).

**Figure 2:**
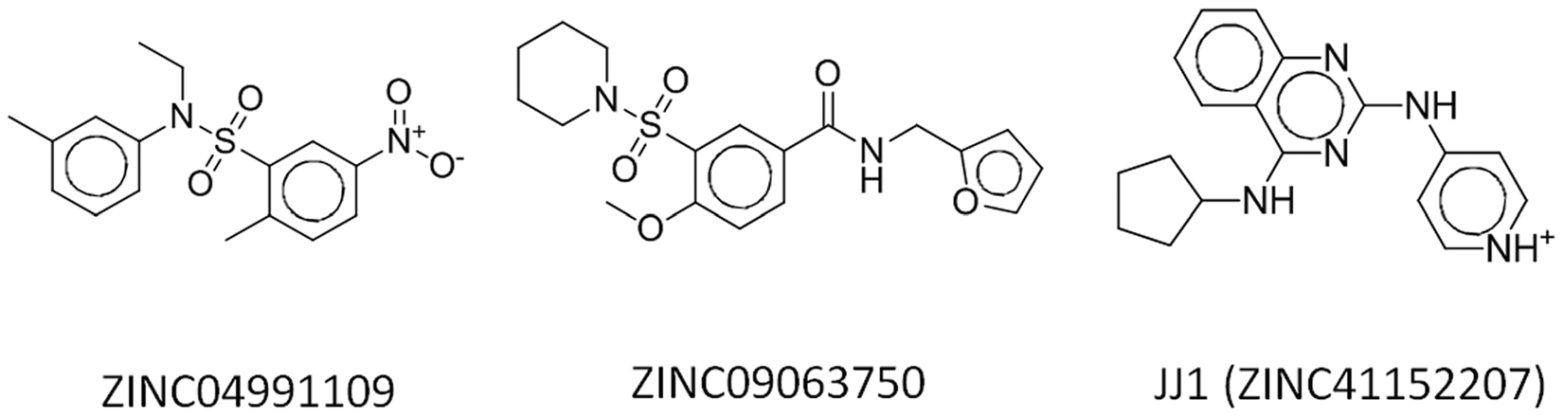
Three molecules showed inhibitory activities in the enzyme-based assay. The degrees of the inhibition in the molecules were more than 50% at the single concentration of 50 μM.

**Figure 3:**
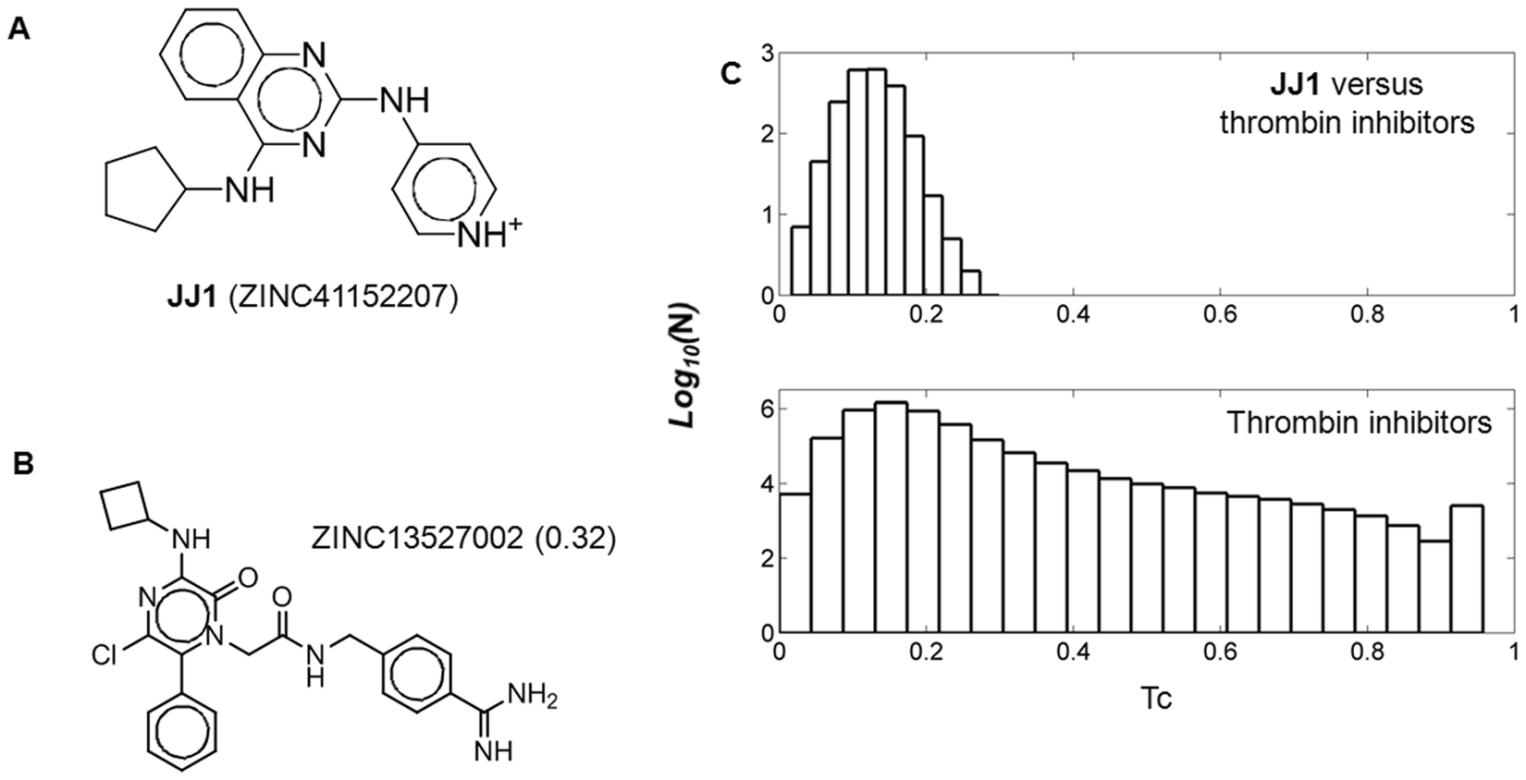
Chemical information of **JJ1** and cheminformatics. (**A**) Chemical structure **JJ1**. The corresponding ZINC ID is written in parentheses. (**B**) The closest chemicals to **JJ1** among the known thrombin inhibitors. Of the non-peptide 2038 thrombin inhibitors registered in the BindingDB database^20^, that showing the highest Tanimoto coefficient (Tc) values with Morgan circular fingerprint from RDKit (http://www.rdkit.org) was ZINC13527002. The values in parentheses indicate the corresponding Tc. (**C**) Histogram analyses of pairwise Tc values. The distributions of Tc values between **JJ1** and the thrombin inhibitors and of those between thrombin inhibitors are drawn. For clarity, the frequencies (y-axis) are scaled logarithmically.

Prior to the high-throughput virtual screening, we selected the best crystal structure for docking with DOCK 3.6^17^ using the selected inhibitors and their physicochemically matched but topologically different decoys. The values of the logarithmically scaled area under the curve (LogAUC) (26.1 ± 10.4%) in the receiver operating characteristic (ROC) curve were more widespread than those of the AUC (70.7 ± 3.6%) in 366 thrombin structures, although the two values of LogAUC and AUC showed correlation with a Pearson’s coefficient of 0.77 (Fig. S1). Because the earlier enrichment of true positives is more important for high throughput SBVS, we used the value of LogAUC as a metric, selecting 2CF9-H^18^ as the best structure. The 2CF9-H structure^18^ resulted in values of 76.1% and 57.4% for the AUC and LogAUC, respectively. Besides, the enrichment at 1% (EF1) was 37.1. The values of LogAUC and EF1 were approximately two-fold higher than the averages (26.1 for LogAUC and 17.1 for EF1) (Figs 1 and S1). Some structures had higher AUC values than that of 2CF9-H, but the value of LogAUC was the highest for 2CF9-H (Fig. S1). The optimization necessitated the calculations of more than 2 million protein and ligand pairs. The high speed of DOCK 3.6 and the in-house Linux cluster system consisting of 128 cores efficiently managed the calculations in three days. Of the candidate molecules having the lowest scores in the high-throughput SBVS, we chose the molecules with desirable geometries and physicochemical properties by a simple manual and visual inspection. For instance, we omitted ZINC08837887 that was ranked as a top candidate with the lowest score, −96.28, because of the noncomplementary fit of the functional moiety. It is known that the scoring function of virtual screening itself is insufficient to capture such an abnormality^19^. Table S1 and Fig. S2 list the top 40 molecules, which were selected through high-throughput SBVS; we purchased these molecules to measure enzymatic inhibitory activity. The subsequent enzyme assay revealed inhibitory effects of three molecules: ZINC04991109, ZINC09063750, and ZINC41152207 at a single dose of 50 μM (Fig. 2). Only ZINC41152207 demonstrated cellular activity; we named the molecule **JJ1** and investigated its activity in detail (Fig. 3).

### Cheminformatics

To verify the novelty of a scaffold of **JJ1** based on chemical similarity, we calculated the pairwise Tanimoto coefficient (Tc) values between **JJ1** and DTIs. After exclusion of DTIs that originated from peptides, we extracted 2038 molecules from the database BindingDB^20^. The average value of Tc was 0.12 ± 0.03 with a maximum value of 0.32 for ZINC13527002. The comparison of the distributions of the Tc values between **JJ1** and 2038 inhibitors reflected the novelty of **JJ1** as a DTI (Fig. 3). The quantified inhibition of ZINC13527002 for thrombin was IC_50_ = 4.6 μM, although its cellular activity was not reported^21^.

**Figure 4:**
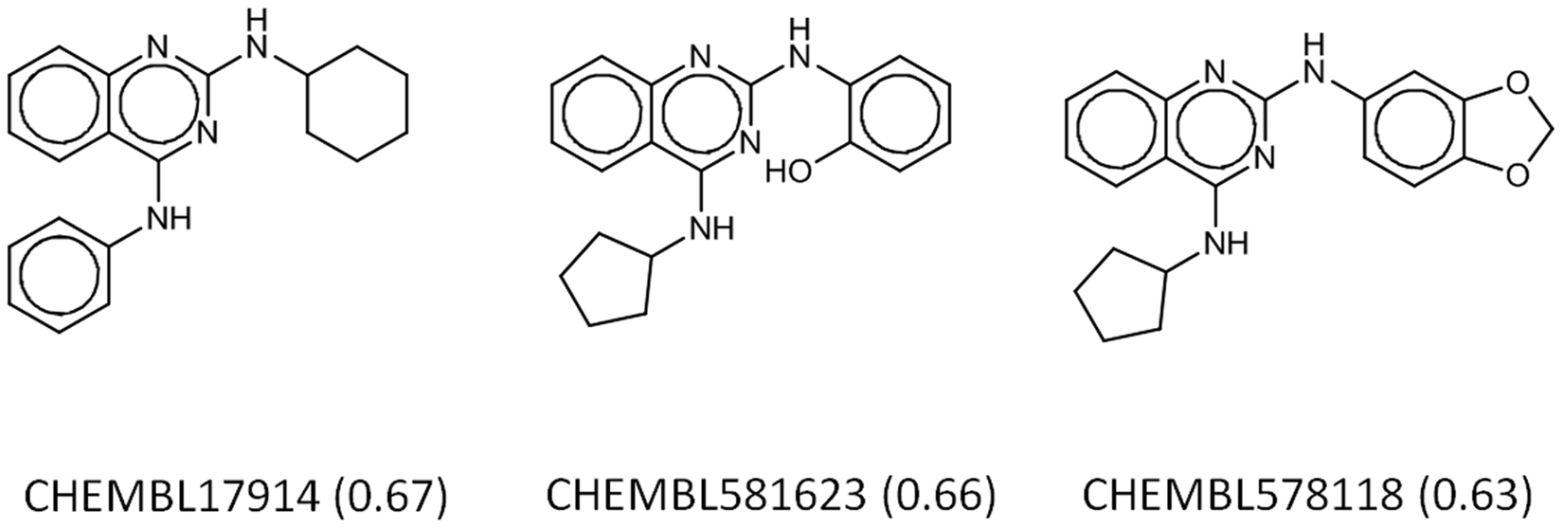
Three analogs of **JJ1** from the ChEMBL database. The molecules showing a higher Tanimoto coefficient (Tc) than 0.6 with Morgan circular fingerprint from the RDKit (http://www.rdkit.org) were extracted. Corresponding Tc values are written in parentheses.

Cheminformatics helps to study the potential bioactivity of the **JJ1** scaffold. There is no deposited activity for **JJ1** in the ChEMBL database^22^. Of the molecules in ChEMBL, the following have similarities higher than 0.6 by the Tc to **JJ1**: CHEMBL17914 (Tc = 0.67), CHEMBL581623 (0.66), and CHEMBL578118 (0.63) (Fig. 4). Although their activities have not been reported in publications, CHEMBL17914 is linked with the inhibition of neuropeptide Y receptor type 5, whereas CHEMBL581623 and CHEMBL578118 have antimalarial effects; apparently, the activities of the compounds are unrelated to antithrombotic activity.

### Predicted binding mode by docking simulation

**Figure 5:**
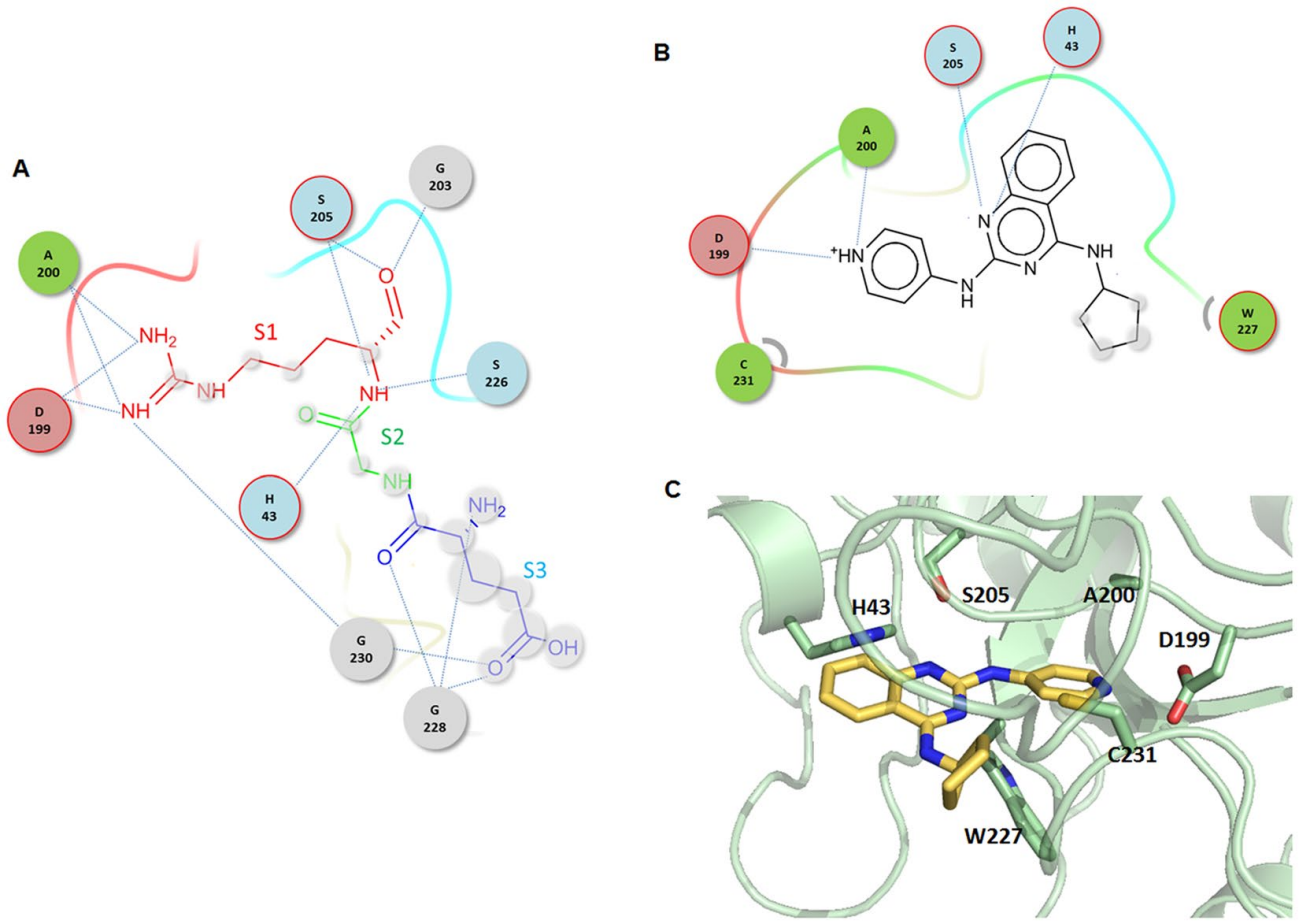
Schematic drawings of proposed binding mode of **JJ1**. Only the residues that participate in intermolecular interactions are drawn with a cutoff of 3.5 Å. (**A**) The substrate, Glu-Gly-Arg, in complex with thrombin^69^ is drawn as a reference, where S1 (red), S2 (green), and S3 (blue) indicate the positions of Arg, Gly, and Glu residues, respectively. Residues in thrombin are drawn in circles, residues in circles with the red outline contact inhibitors with side chains, while the others employ backbone atoms. Atoms exposed to solvent in inhibitors are shaded in grey. Dashed lines correspond to hydrophilic interactions. Curves in residues indicate hydrophobic contacts. (**B**) Binding mode of **JJ1**, symbols and colors as for (**A**). (**C**) Three dimensional illustration of binding pose.

Figure 5 presents the binding mode of **JJ1** as predicted by the docking simulations. The reliability of the model could be confirmed by comparing the pose with those of docked JJ1 in other thrombin crystal structures. The current mode was the most populated when JJ1 was docked into all crystal structures corresponding to 30% (=109/366) of all the docked poses within the 2 Å root mean square deviation (RMSD) cut-off. The existence of the arginine at the P1 position of a substrate is important for interacting with thrombin. The specific cleavage of a peptide bond of a natural substrate occurs at the carbonyl group of the Arg. A bidentate salt bridge between the amidine of Arg at the P1 position of the substrate and the carboxylate of Asp-199 at the S1 position of thrombin plays a central role in fixing the substrate. Many DTIs possess the amidine moieties that anchor DTIs to Asp-199 at the S1 position. In contrast, **JJ1** occupies the S1 position of thrombin with a 4-pyridyl moiety. The key interactions by **JJ1** exploited the side chains of the His-43 and Asp-199 residues; the residues Ala-200, Ser-205, Trp-227, and Cys-231 also contributed to the interactions with **JJ1**. In order to determine whether the contacts observed in our models were common in DTIs, we searched for complex structures within thrombin and inhibitors. Of the 297 inhibitors found in the PDB database, 249 interact with His-43 and 185 interact with Asp-199. This indicates that the binding modes found in **JJ1** are widespread in thrombin inhibitors.

**Figure 6:**
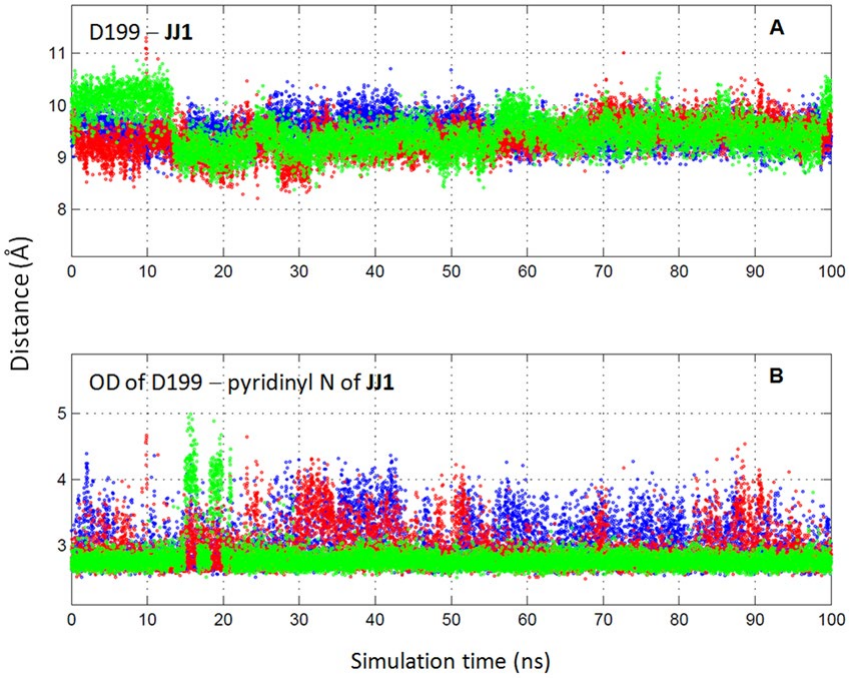
MD trajectories of **JJ1** in complex with thrombin. (**A**) Time evolutions of the center of mass distances between Asp-199 and **JJ1**. (**B**) Time evolutions of the hydrogen bond distances between OD of Asp-199 and pyridinyl N of **JJ1** atoms. Each color indicates an independent trajectory started from a structure with a different random seed.

The predicted binding modes were further assessed by molecular dynamics (MD) simulation. We repeated 100 ns MD simulations three times from the docked model of **JJ1** with the different random seeds. The overall folds in both **JJ1** and thrombin showed no significant change compared with the starting structure during the period. The distance between Asp-199 and **JJ1** in the center of mass was kept as 9.44 ± 0.32 Å in the three trajectories (Fig. 6A). Of the key intermolecular interactions through the His-43 and Asp-199 residues, the more dominant interaction in the simulations was the intermolecular hydrogen bond with Asp-199. The occupancies of the hydrogen bond in the three trajectories were 94, 75, and 63%, respectively, which produced the time-averaged distance of 2.91 ± 0.31 Å between the pyridinyl N of **JJ1** and the carboxylic OD of the Asp-199 atoms (Fig. 6B). In contrast, the interaction through His-43 was more divergent dependent on the trajectory. The averaged distances between the CE1 of His-43 and the carbon in the cyclopentyl moiety of **JJ1** were 5.38 ± 1.20, 4.30 ± 1.00, and 3.74 ± 0.37 Å. The result does not indicate an insignificant contribution of the cyclopentyl moiety to the intermolecular interaction. It is noteworthy that the van der Waals term (−33.26 kcal/mol) constitutes over half of the DOCK 3.6 scoring function of **JJ1** (−56.01). In order to clarify the role of the cyclopentyl moiety, we performed MMPBSA analyses using **JJ1** and its two analogs, **JJ1a** and **JJ1b** (Fig. S4). The cyclopentylamino group is removed for **JJ1a** and replaced by the propylamino moiety for **JJ1b**. MMPBSA calculates the end-state free energy upon forming a protein-ligand complex in solution by a post-processing method^23,24^. The mean (±standard deviation, SD) values of MMPBSA-derived free energy for **JJ1**, **JJ1a**, and **JJ1b** in three independent runs with different random seeds were −19.7 (±3.3), −5.0 (±2.6), and −9.2 (±2.8) (kcal/mol), respectively. This demonstrates the substantial role of the cyclopentyl moiety in **JJ1** for interacting with thrombin, at least in a qualitative way. The redesign of the cyclopentyl part using computational simulation may be a good strategy to prepare a more potent molecule from **JJ1**.

### Enzyme inhibitory effect of JJ1 against different human enzymes

The results of a chromogenic substrate assay demonstrated that **JJ1** was a potent inhibitor of human thrombin with *K*_i_ = 0.019 μM (Table 1). The selectivity of **JJ1** for thrombin compared to that for other human enzymes is shown in Table 1. **JJ1** is highly selective for thrombin and demonstrated a selectivity ratio, based on the respective *K*_*i*_ values, of >10,000 for thrombin versus trypsin, factor Xa, elastase, plasmin, protein Ca, streptokinase, tPA, and urokinase. Further, it was more potent than argatroban (*K*_i_ = 0.025 μM) and less potent, but more selective, than efegatran (*K*_i_ = 0.017 μM) (Table 1). This was most notable for trypsin, which was inhibited at lower concentrations of efegatran than those reported for thrombin. Efegatran also inhibited plasmin, protein Ca, streptokinase, tPA, and urokinase; the selectivity ratios for these compounds were 28–1470 versus those for thrombin.

**Table 1:** Enzyme kinetics and selectivity of **JJ1** against different human enzymes.

Enzyme	JJ1		Efegatran		Argatroban	
	*K*_*i*_^a^	Selectivity ratio^b^	*K*_*i*_	Selectivity ratio	*K*_*i*_	Selectivity ratio
α-Thrombin	0.019 ± 0.0005 (IC_50_ = 0.15)^c^	1	0.017 ± 0.0003 (IC_50_ = 0.025)	1	0.025 ± 0.0011 (IC_50_ = 0.12)	1
Trypsin	>250	>20,000	0.011 ± 0.0041	0.65	4.2 ± 0.5	168
Factor IXa	>250	>10,000	ND	ND	ND	ND
Factor Xa	>250	>10,000	92 ± 9	5411	>250	>10,000
Factor XIa	>250	>10,000	ND	ND	ND	ND
Elastase	>250	>10,000	>250	>14,000	>250	>10,000
Plasmin	>250	>10,000	0.48 ± 0.04	28	>250	>10,000
Protein Ca	>250	>10,000	1.2 ± 0.15	70	>250	>10,000
Streptokinase	>250	>10,000	1.4 ± 0.11	82	>250	>10,000
tPA	>250	>10,000	1.5 ± 0.3	88	>250	>10,000
Urokinase	>250	>10,000	2.5 ± 0.3	147	>250	>10,000

^a^ *K*_*i*_ is represented as by the mean ± SD (n = 5), μM. ^b^Selectivity ratio = *K*_*i*_ enzyme/*K*_*i*_ α-thrombin. ^c^IC_50_ is presented in μM. ND, not determined.

### Effects of JJ1 on the activity and production of thrombin

**Figure 7:**
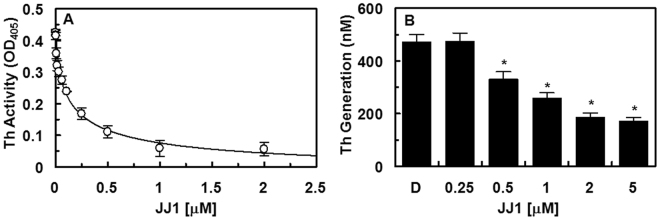
Effects of **JJ1** on the inactivation and production of thrombin. (**A**) Inhibition of thrombin (Th) by **JJ1** was measured using a chromogenic assay, as described in the “Materials and Methods” section. (**B**) The HUVEC monolayer was pre-incubated with FVa (100 pM) and FXa (1 nM) for 10 min with the indicated concentrations of **JJ1**. Prothrombin was added at a final concentration of 1 μM, and prothrombin activation was determined after 30 min, as described in the “Materials and Methods” section. *P < 0.05 vs DMSO (**B**).

First, we determined the anti-thrombin functions of **JJ1** by measurement of the inhibition of thrombin activity using chromogenic substrates. Treatment with **JJ1** resulted in a dose-dependent inhibition of the amidolytic activity of thrombin (IC_50_ = 0.15 μM), which indicated that **JJ1** directly inhibited thrombin activity (Fig. 7A). In a previous study, Sugo *et al*. reported that endothelial cells supported prothrombin activation by FXa^25^. In the current study, pre-incubation of HUVECs with FVa and FXa in the presence of CaCl_2_, before the addition of prothrombin, resulted in thrombin production (Fig. 7B). In addition, treatment with **JJ1** inhibited the production of thrombin in a dose-dependent manner (Fig. 7B).

### Effect of JJ1 on clotting and bleeding time

Incubation of human plasma with **JJ1** also affected the blood coagulation cascade. The anticoagulant activity of **JJ1** was evaluated using aPTT and PT assays and human plasma (Table 2). As shown in Table 2, the aPTT and PT were significantly prolonged by **JJ1**. At 1.66 μM and 1.61 μM, **JJ1** doubled the clotting time in the aPTT and PT assays, respectively. Therefore, our results indicated that **JJ1** inhibited the blood coagulation pathway. To confirm these *in vitro* results, we determined *in vivo* tail bleeding times. The average circulating blood volume of a mouse is 72 mL/kg^26^. The average weight of a mouse used in this study was 27 g and the average blood volume was 2 mL; therefore, the amount of **JJ1** (0.31, 0.61, 1.23 or 3.06 μg per mouse) equaled a peripheral blood concentration of approximately 0.5, 1.0, 2.0, or 5.0 μM, respectively. As shown in Table 2, the tail bleeding times were significantly prolonged by i.v. injection of **JJ1** in comparison to those of the control. The anticoagulation effect of **JJ1** was observed *ex vivo* in mice as demonstrated by the dose-dependent prolongation of the aPTT and PT (Table 3).

**Table 2:** Anticoagulant activities of **JJ1** ^a^.

*In vitro* coagulant assay					
Sample	Dose	aPTT (s)	PT (s)		PT (INR)
Control	saline	23.7 ± 0.5	11.9 ± 0.4		1.00
**JJ1**	0.05 μM	23.5 ± 0.4	12.1 ± 0.2		1.04
	0.1 μM	23.3 ± 0.2	11.7 ± 0.4		0.96
	0.2 μM	24.2 ± 0.4	12.7 ± 0.5		1.17
	0.5 μM	32.4 ± 0.6*	15.4 ± 0.4*		1.86*
	1.0 μM	41.3 ± 0.4*	21.4 ± 0.5*		4.09*
	2.0 μM	50.4 ± 0.6*	25.8 ± 0.3*		6.41*
	5.0 μM	51.6 ± 0.4*	26.5 ± 0.4*		6.83*
Efegatran	5.0 μM	44.2 ± 0.6*	19.2 ± 0.4*		3.15*
Argatroban	5.0 μM	61.4 ± 0.8*	27.4 ± 0.7*		7.40*
Heparin	3.0 IU/mL	82.9 ± 1.2*	24.6 ± 0.4*		5.71*
***In vivo*** **bleeding time (i.v. injection)**					
**Sample**	**Dose**	**Tail bleeding time (s)**		**n**	
Control	Saline	30.2 ± 1.0		5	
**JJ1**	0.31 μg/mouse	30.6 ± 0.8		5	
	0.61 μg/mouse	37.2 ± 1.0*		5	
	1.23 μg/mouse	44.8 ± 1.2*		5	
	3.06 μg/mouse	52.2 ± 1.4*		5	
Efegatran	4.17 μg/mouse	47.4 ± 1.1*		5	
Argatroban	5.08 μg/mouse	62.2 ± 0.8*		5	
Heparin	6.00 IU/mouse	85.4 ± 1.2*		5	

^a^Each value represents the means ± SD (n = 5). *P < 0.05 as compared to control.

**Table 3:** *Ex vivo* coagulation time of **JJ1** ^a^.

Sample	Dose	aPTT (s)	PT (s)	PT (INR)
Control	Saline	32.1 ± 0.4	12.0 ± 0.3	1.00
**JJ1**	0.31 μg/mouse	31.9 ± 0.6	12.4 ± 0.5	1.08
	0.61 μg/mouse	40.1 ± 1.1*	17.2 ± 1.4*	2.37
	1.23 μg/mouse	49.4 ± 1.5*	22.3 ± 0.6*	4.42*
	3.06 μg/mouse	55.8 ± 1.2*	26.8 ± 0.4*	6.88*

^a^Each value represents the means ± SD (n = 5). *P < 0.05 as compared to control.

### Effects of JJ1 on clot-bound thrombin activity

**Figure 8:**
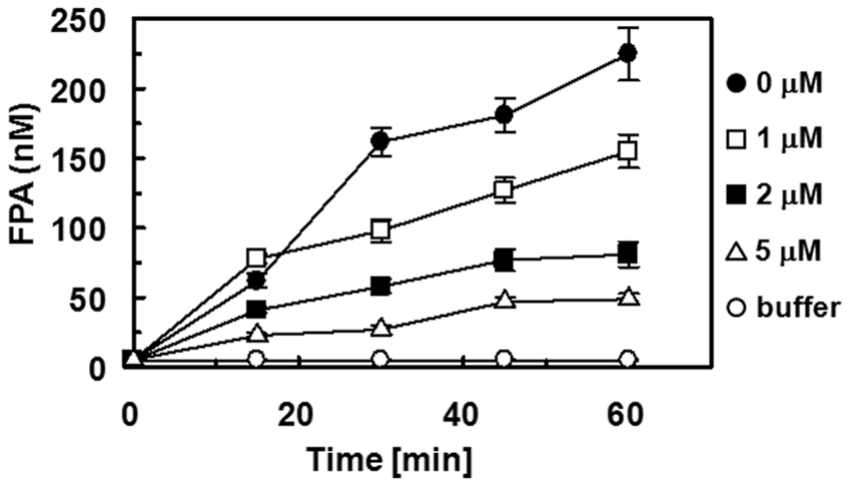
Effects of **JJ1** on FPA generation in purified fibrinogen solution by clot-bound thrombin. Purified fibrinogen (2 mg/mL) in TBS was overlaid on the washed clots in the presence or absence of **JJ1** (0–5 μM) and aliquots were removed at different time points for measurement of FPA by ELISA. The data represent the mean ± SD of three independent experiments performed in triplicate.

To determine if **JJ1** effectively inhibited clot-bound thrombin, an *in vitro* clot-bound thrombin assay system was used. Fibrin clots were formed by recalcification of citrated plasma and washed extensively to remove any FPA trapped within the clots. Plasma was then placed over the washed clots and FPA generation was determined. There was a time-dependent generation of FPA in plasma that was not caused by the release of FPA trapped within the washed clots, because the addition of buffer over the clots produced only a minimal amount of FPA (5.25 ± 0.35 nM at 60 min) (Fig. 8). The endogenous FPA level was 5.05 ± 0.31 nM in these studies. The data showed that fibrin clots retained a significant amount of active thrombin that was capable of interaction with plasma fibrinogen to generate FPA. A substantial amount of FPA was generated in the supernatant (225 ± 19.2 nM at 60 min of incubation), which was inhibited by **JJ1** in a dose-dependent manner (Fig. 8, IC_50_ = 2.45 μM).

### Effect of JJ1 on fibrin polymerization, platelet aggregation, and cellular viability

**Figure 9:**
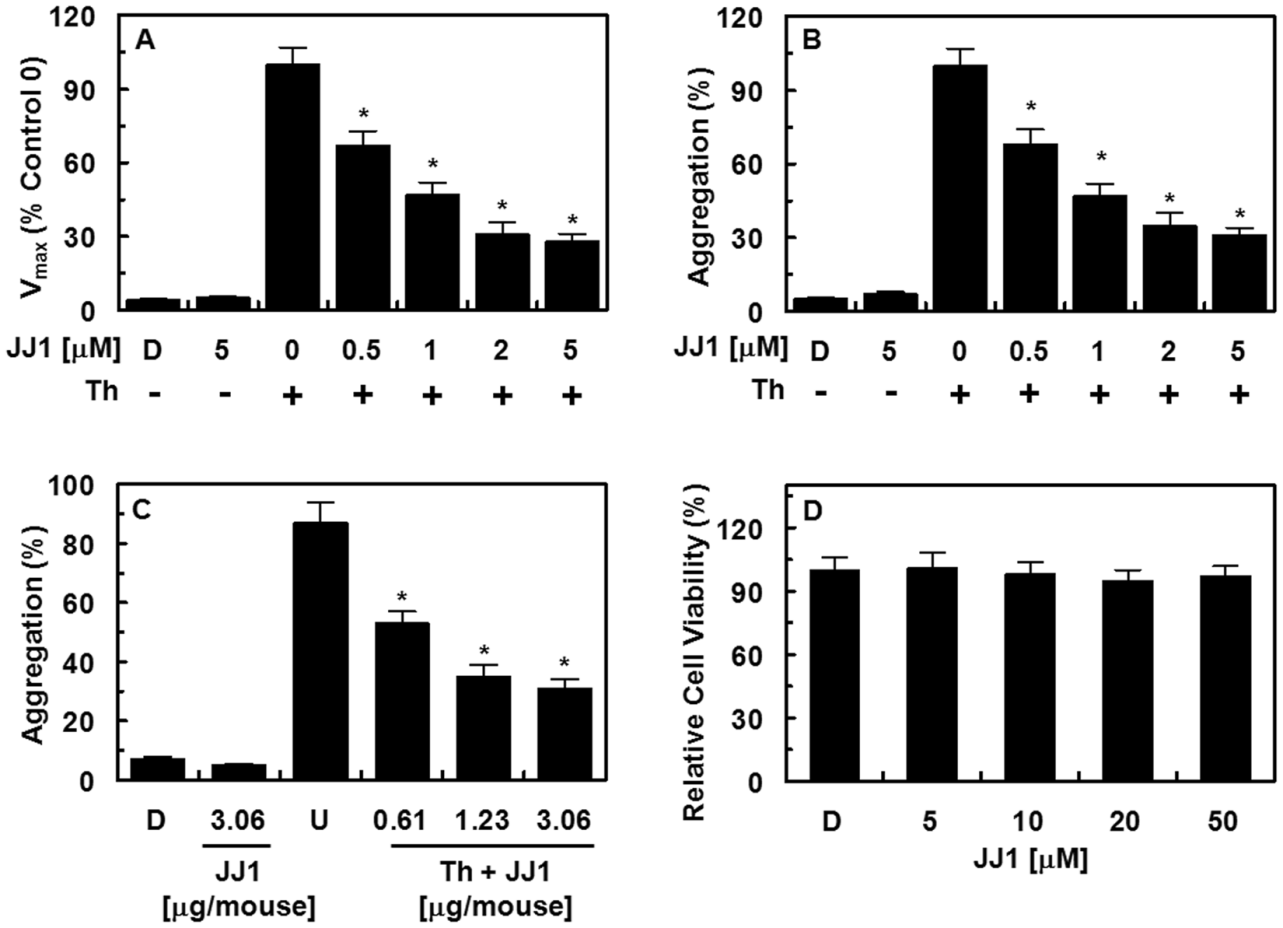
Effects of **JJ1** on fibrin polymerization, platelet aggregation, and its cytotoxicity. (**A**) Thrombin (Th)-catalyzed fibrin polymerization was monitored at the indicated concentrations of **JJ1** using a catalytic assay, as described in the “Materials and Methods” section. The results are expressed as the V_max_ values as a percentage of the controls. (**B**) The effect of **JJ1** on human platelet aggregation induced by thrombin. (**C**) **JJ1** in DMSO was injected intravenously at the indicated concentration. The effects of **JJ1** on mouse platelet aggregation induced by thrombin were monitored *ex vivo*. (**D**) The effect of **JJ1** on cellular viability was measured using the MTT assay. D = 0.2% DMSO used as the vehicle control. The data represent the mean ± SD of three independent experiments performed in triplicate. *P < 0.05 vs Th alone.

The effect of **JJ1** on thrombin-catalyzed fibrin polymerization in human plasma was determined from the changes in the absorbance at 360 nm, as described in the Materials and Methods section. The incubation of human plasma with **JJ1** significantly decreased the maximal rate of fibrin polymerization (Fig. 9A). To eliminate the effect of the sample pH, all dilutions were made in 50 mM TBS at pH 7.4. We also evaluated the effect of the same volume of DMSO on human plasma and found that this did not affect coagulation. To confirm the anticoagulant activities of **JJ1**, a thrombin-catalyzed platelet aggregation assay was performed. As shown in Fig. 9B, treatment with **JJ1** significantly inhibited human platelet aggregation induced by thrombin in a concentration-dependent manner. These *in vitro* results were confirmed in an *ex vivo* platelet aggregation assay (i.v. injection, Fig. 9C). These results were consistent with the antithrombin assay and therefore suggested that the antithrombotic mechanism underlying the action of **JJ1** involved the inhibition of fibrin polymerization, the inhibition of the activity and production of thrombin, or both. Furthermore, as determined using the MTT assay in HUVECs treated with the compounds for 24 h, no compound affected cell viability at concentrations up to 50 μM (Fig. 9D). The pharmacokinetic analyses of injected **JJ1** are shown in Table 4. When mice in a fasting state were injected with **JJ1** (2 mg/kg), the mean value of the AUC was 6.1 μg·h/mL and the half-life was 3.1 h.

**Table 4:** Pharmacokinetic parameters of **JJ1** after i.v. injection in male C57BL/6 mice.

Parameters	JJ1 (2 mg/kg)
AUC^a^	6.1 μg∙h/mL
Half-life	3.1 h

^a^AUC = area under curve.

### *In vivo* effects of JJ1 in an arterial thrombosis and a pulmonary thrombosis model

**Figure 10:**
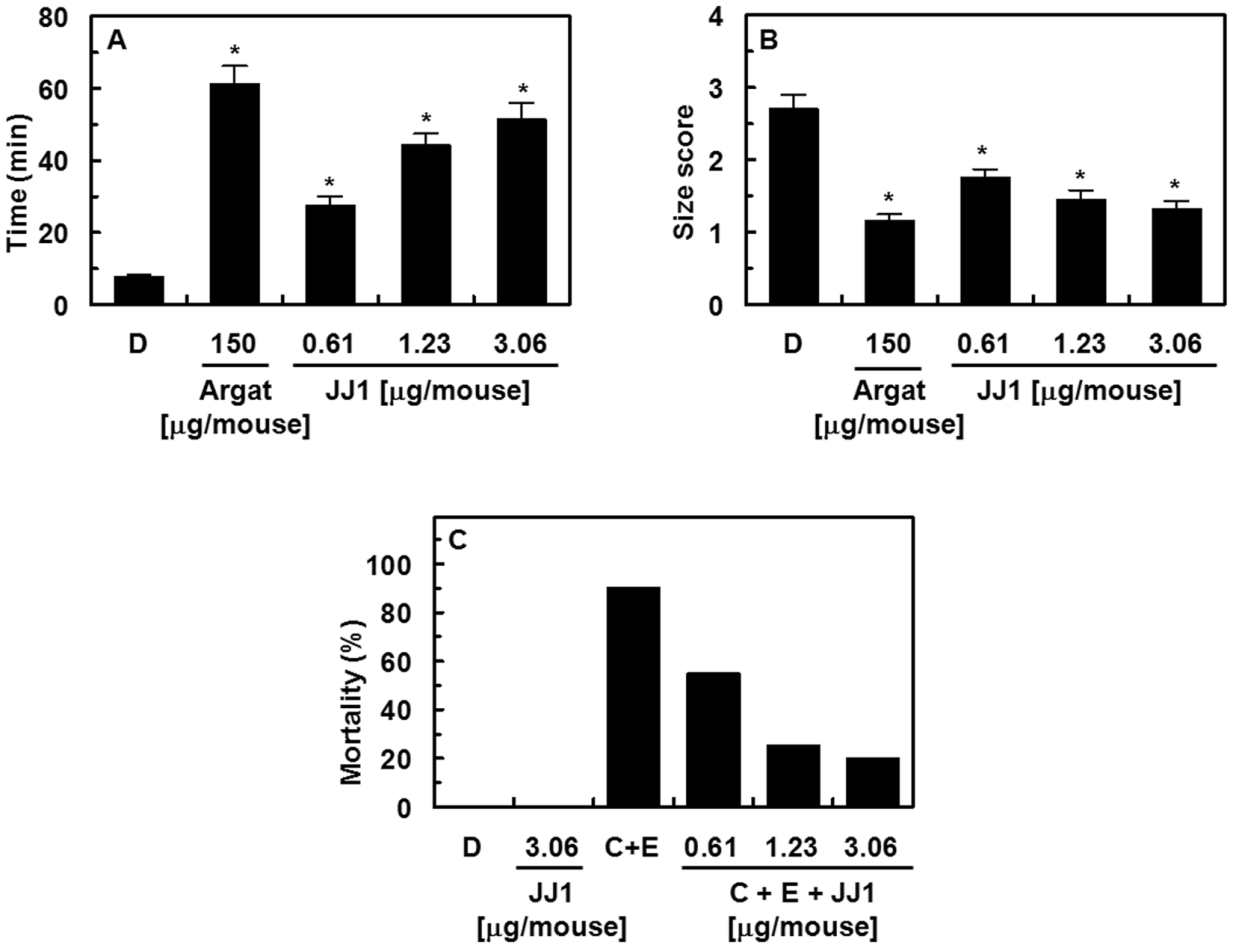
Effects of **JJ1** on arterial thrombosis and on acute thrombosis. (**A**) Time to large thrombus formation by **JJ1**. Argatroban (150 μg/mouse, Argat) was used as the positive control. (**B**) The size score of the thrombus at 60 min after FeCl_3_-treatment, measured as described in “Materials and Methods”. (**C**) After **JJ1** was injected intravenously, a mixture of collagen (**C**, 500 μg/kg) plus epinephrine (E, 50 μg/kg) was injected into the tail vein of mice to induce acute thrombosis 1 h later. Then, groups of mice (n = 20) were carefully examined for 15 min to determine whether the mouse was paralyzed, dead, or had recovered from the acute thrombotic challenge. D = 0.2% DMSO used as the vehicle control. *P < 0.05 vs DMSO.

A mouse model of ferric chloride (FeCl_3_)-induced carotid artery thrombosis^27^ has been commonly used to assess antiplatelet effects. The time required for thrombus formation and the size of the resulting thrombi are summarized in Fig. 10. The data showed that endothelial injury after FeCl_3_ treatment in control mice led to the growth of large thrombi after 7.7 ± 0.6 min. **JJ1** significantly slowed the growth of thrombi (Fig. 10A). We further examined the effect of **JJ1** on thrombus size at 60 min after FeCl_3_-induced endothelial injury (Fig. 10B). The results showed that **JJ1** reduced FeCl_3_-induced thrombus formation. Although argatroban (150 μg/mouse) treatment resulted in much smaller (1.15 ± 0.12) and slower (61.2 ± 5.1 min) thrombus size than did **JJ1** (3 μg/mouse), there was a 50-fold difference in the concentration of **JJ1**. In addition, the results of the *in vivo* pulmonary thrombosis model are shown in Fig. 10C. An intravenous injection of a mixture of collagen and epinephrine to mice induced massive pulmonary thrombosis that caused acute paralysis and ultimately led to sudden death (90–95% mortality). The mortalities in **JJ1-**treated groups decreased significantly compared with those in the collagen- and epinephrine-treated groups (Fig. 10C).

The safety of **JJ1** is another promising feature. Animal safety markers such as aspartate transaminase (AST) and alanine transaminase (ALT) (markers of hepatic injury) and creatinine and blood urea nitrogen (BUN) (markers of renal injury) were assessed with blood samples obtained at 48 h after **JJ1** injection. The results showed that there were no significant differences in the plasma levels of AST, ALT, BUN, and creatinine after 2 days compared to those in DMSO-treated animals (Table 5).

**Table 5:** Biochemical safety parameters of male C57BL/6 mice after treatment with **JJ1** ^a^.

Parameters	DMSO	JJ1 (3.06 μg/mouse)
ALT (IU/L)	7.9 ± 0.53	7.5 ± 0.63
AST (IU/L)	14.7 ± 1.32	15.1 ± 1.29
Creatinine (mg/dL)	0.121 ± 0.023	0.139 ± 0.012
BUN (IU/dL)	16.3 ± 1.57	17.1 ± 1.31

^a^Each value represents the means ± SD (n = 5).

## Discussion

Despite extensive research effort and the subsequent identification of an impressive number of high affinity thrombin inhibitors, the clinical use of parenterally available preparations of thrombin inhibitors is still limited. Various attributes make inhibitors unattractive for clinical applications. A considerable number of thrombin inhibitors have problems including poor selectivity, inherent toxicity, high-plasma protein binding, poor metabolic stability, rapid elimination from the blood, low anticoagulant activity, and poor oral bioavailability.

In this study, a computational protocol that selects the best structure for high-throughput SBVS by using the known inhibitors and their property-matched decoys resulted in a new type of thrombin inhibitor. Whether the selection process may have influenced the chemical structures of the candidates should be addressed. Although the 92 inhibitors used to select the best structure included 60 molecules with amidine moieties, only one molecule of 40 candidates had the arginine-like amidine group. This suggests that the inhibitors used to select the template structure were unlikely to bias the diversity of the candidates. Our data showed that the enrichments of the true positives depended heavily on the template structures though they share apparent close similarities. This demonstrates the advantage of using an ensemble structure. If the experimental structures are limited in number, the use of an ensemble by molecular dynamics simulation is a good alternative^16^.

Thrombin plays a pivotal role in the pathogenesis of arterial and venous thrombosis. It is the principal component responsible for maintenance of an intricate balance between the interactive elements of the vessel wall, coagulation factors, and platelets. A potent, selective, and highly efficacious inhibitor of thrombin has the potential to be a highly effective antithrombotic drug in both arterial and venous environments. In this study, we described the antithrombotic properties of a new thrombin inhibitor, **JJ1**. It should be noted that many known thrombin inhibitors isolated from animals have a molecular weight in the 0.5–10 kDa range, or greater, and the molecular weight of **JJ1** (306.4) was relatively smaller than any other known thrombin inhibitors; therefore, **JJ1** has the advantages of low immunogenicity and low production cost when compared with relatively large antithrombotic proteins or peptides. The compound is an inhibitor of thrombin that binds to thrombin with a *K*_*i*_ of 0.019 μM. **JJ1** is highly selective against other serine proteases of the coagulation cascade and the fibrinolytic system. Additionally, the mean value of the AUC and half-life of **JJ1** were 6.1 μg·h/mL and 3.1 h, respectively.

Thrombin action on fibrinogen involves limited proteolysis, which results in the production of the fibrin monomer and the fibrinopeptides A and B. A number of attempts have been made to develop tests for thrombin action based on the detection of the fibrin monomer^28–31^. Thrombin cleaves arginyl-glycine bonds to release FPA (16 amino acids) from the NH_2_-terminal segment of the Aα chain and fibrinopeptide B (14 amino acids) from the NH_2_-terminal segment of the Bβ chain^28–31^. Using the plasma FPA levels as an index of thrombin activity, the FPA assay was used as a technique for studies of the inhibitory ability of **JJ1** to block FPA release. This result supported the importance of **JJ1** in the close relationship with the anti-thrombotic effects of **JJ1** against thrombin.

Thrombus formation is the result of two main processes. Initially, platelets are activated and aggregate after exposure to stimulants during vascular injury. This process is followed by the initiation of coagulation, which leads to the formation of thrombin and subsequent fibrin network deposition. Thrombin is recognized as a highly potent stimulator of platelets and plays a major role in the formation of arterial thrombi. Therefore, thrombin inhibitors should demonstrate potent inhibition of thrombin generation and platelet aggregation. In this study, **JJ1** inhibited the production of thrombin in HUVECs and thrombin-induced aggregation in a human platelet preparation. This *in vitro* activity translated to *in vivo* antithrombotic activity in models of *ex vivo* platelet aggregation, arterial thrombosis, and pulmonary thrombosis.

The pre-clinical evaluation of the antithrombotic potential of novel molecules requires the use of reliable and reproducible experimental animal preparations of thrombosis. One of the most widely used procedures employs topical application of ferric chloride (FeCl_3_) to an artery^32,33^. The mouse model of FeCl_3_-induced carotid artery thrombosis is an arterial thrombosis and a pulmonary thrombosis model and one of the most established and commonly used preparations to determine the efficacy of novel antithrombotic drugs *in vivo* ^27^. This is a simple and well-established model known to be sensitive to both anticoagulant and antiplatelet drugs^32,33^. The mouse model of acute arterial thrombosis that was used in our experiment, which was developed by the application of FeCl_3_ to the outer tissues of arteries that had lost endothelial cell protection owing to circulating platelets and components of the coagulation cascade, has been used to find both anticoagulant and antiplatelets drugs^34,35^. A hallmark of the FeCl_3_-injury model is its sensitivity to thrombin inhibitors^36^. The role of platelets in this model was similar to the platelet/platelet interaction in the human arterial thrombotic process. In these studies, we showed a dose-dependent reduction in thrombus formation during intravenous administration of **JJ1**. **JJ1** resulted in a significant reduction in the growth and formation of thrombi.

The PT, aPTT, fibrin polymerization, and platelet aggregation methods are most commonly used to determine the efficacy of novel anti-thrombotic drugs^37,38^. At the maximum antithrombotic dose, **JJ1** resulted in 2.17-fold and 2.22-fold prolongation of aPTT and PT, respectively. In contrast, the dose of efegatran needed to achieve the maximum antithrombotic effect resulted in a 1.86-fold and 1.61-fold prolongation of aPTT and PT, respectively. In addition, compounds **1** and **2** caused a significant decrease in the maximal rate of fibrin polymerization and inhibited platelet aggregation. The quantity of systemic anticoagulation that will be tolerated in a regimen of antithrombotic therapy remains to be determined.

As shown in several studies, inflammation and coagulation are closely related processes that may have considerable effects on each other^39,40^. This is most apparent in platelet activation, fibrin formation, and resolution, as well as in the physiological anticoagulant cascades^39,40^. From our data, we hypothesized that **JJ1** was a promising novel anti-inflammatory mediator owing to its inhibitory effects on coagulation; however, well-designed prospective studies are warranted to prove this hypothesis. Heparin has been used as a commercial anticoagulant for the prevention of venous thromboembolic diseases for more than 60 years^41,42^. However, heparin has side effects, such as the inability to inhibit fibrin-bound thrombin activity, ineffectiveness in congenital or acquired antithrombin deficiencies, development of thrombocytopenia, increased risk of thromboembolic disease if the therapeutic response is not achieved, and increased risk of bleeding if the therapeutic range is exceeded^42,43^. Furthermore, bovine lung and pig intestine, which are the primary sources from which heparin is extracted, only contain low amounts of available material^43^. Therefore, the need to discover alternative sources of anticoagulants has arisen from the demand for safer anticoagulant therapy. Based on the current findings, if the beneficial therapeutic effects can be established, **JJ1** will provide a new chemotype for the development of anticoagulants.

## Conclusion

**JJ1** is a potent inhibitor of thrombin that demonstrates selectivity over other biologically important serine proteases. *In vitro* and *in vivo* studies have shown that **JJ1** is also a potent inhibitor of thrombin-induced platelet aggregation. **JJ1** demonstrated potent antithrombotic efficacy in arterial thrombosis and a pulmonary thrombosis model via the corresponding clotting time. Our data indicated that direct thrombin inhibitors such as **JJ1** may present an improved option for the treatment of various thrombotic disorders.

## Materials and Methods

### Reagents

Factor Va, Xa, prothrombin, and thrombin were obtained from Haematologic Technologies (Essex Junction, VT). The aPTT assay reagent and PT reagents were purchased from Fisher Diagnostics (Middletown, VA). Argatroban was purchased from Santa Cruz Biotechnology, Inc. (Dallas, TX). Inhibitors predicted by high-throughput virtual screening were purchased from ChemBridge (San Diego, CA). All other reagents were of the highest commercially available grade.

### Structure-based high-throughput virtual screening and cheminformatics

DOCK 3.6^17^ was selected as an engine for SBVS, because it could faithfully reproduce the poses of inhibitors found in x-ray crystal structures (Fig. 1). Of the available 366 thrombin structures deposited in the PDB database, the structure (PDB ID: 2CF9) that showed the most favorable metrics in test runs with the 96 known inhibitors and their physicochemically matched 5398 decoys was employed as a template. The representative inhibitors were selected from the database BindingDB^20^ to cover the chemical structures of non-peptide DTIs. After extracting all human thrombin inhibitors with molecular weights smaller than 500 Da and activities stronger than 10 μM by *K*_*i*_ or IC_50_ from BindingDB, the molecules were ordered according to their ligand efficiency (LE) values. The value of LE is defined by −1.37 × Log_10_ *K*_i_/HA, where HA is the number of hetero atoms. If the IC_50_ value was available instead of the *K*_i_, the *K*_i_ was assumed to be IC_50_/2. Then, only the molecules having the larger LE values, but not sharing similarity higher than 0.6 in terms of Tanimoto coefficient (Tc) with other molecules, were selected as the representatives. Peptide DTIs were removed by visual inspection. The DUD-E server produced the decoys^44^. All thrombin structures were overlaid using TM-align^45^ to have the same orientation and ligand binding sites, which facilitated the preparation of the proteins for docking. The coordinates of the substrate found in x-ray structures guided the positions of the spheres and grids that the new inhibitor bound to, and in the calculation of intermolecular energy. Explicit solvents were omitted. Each protein was handled as a rigid body. The parameters and protocol for SBVS were adapted from DOCK-Blaster^46^ (Fig. S3). Small molecules with flexibase formats and ligand desolvation scoring terms (Δ*G*_desolv_) were extracted from the ZINC database^47^. The values of ligand sampling parameters for bin size, bin size overlap, and distance tolerances were 0.2 Å, 0.1 Å, and 1.2 Å, respectively. The scoring function (*E*_score_) was calculated by summation of the intermolecular electrostatics (*E*_elec_) and van der Waals (*E*_vdw_) energies adjusted by ligand desolvation (Δ*G*_desolv_)^17^ as *E*_score_ = *E*_elec_ + *E*_vdw_ + Δ*G*_desolv_. The electrostatic and van der Waals energies were calculated based on the Poisson-Boltzmann calculations by Delphi^48^ and the AMBER united-atom force field^49^, respectively. The best structure was determined based on three metrics: area under the curve (AUC)^50^, logarithmically scaled AUC (LogAUC)^17^, and the enrichment factor at 1% (EF1)^51^. The proportions of each molecule as false-positive and true-positive were plotted as *x* and *y* variables to form the receiver operating characteristic (ROC) curve in 2D. The AUC of the ROC curve has a range of 0–1. Logarithmical scaling of the *x*-axis of the AUC in the range of 0.001–1 to −3–0 expands the area to 3. Upon dividing the value of the area by 3 for normalization and subtracting 0.145, the expected enrichment due to chance follows, defined as LogAUC. The value of LogAUC lies in the range of −0.145 to 0.855. In this study, the AUC and LogAUC were expressed as percentages. The EF1 was defined as the enrichment factor in 1% of compounds. For instance, an EFI value of 10 indicates that 10% of the true positives are recovered by the time 1% of the compounds are screened. High-throughput SBVS docked approximately 400,000 small molecules from the ChemBridge Express virtual library with the selected structure as a template. Of the original 505,000 molecules, we selected those that had desirable drug-like properties based on Lipinski’s rule of five^52^. A Tc with Morgan circular fingerprint generated by RDKit (http://www.rdkit.org) was used to quantify the similarity between molecules. Tc values range between 0 and 1, where 0 and 1 correspond to no overlap and perfect overlap of the two compounds, respectively.

### Molecular dynamics simulation

The missed residues in the structure for SBVS were filled using Modeller (ver 9.8)^53^. The SQM and LEaP programs in AMBER 16^54^ generated the GAFF force field of **JJ1** for the molecular dynamics (MD) simulation, with the assumption of a net charge of +1, whereas the ff14SB force field was employed for thrombin. The TIP3P water system, which included Na^+^ and Cl^−^ ions for neutralization, solvated the complex structure in an octahedral box, which was kept at least 10 Å from the protein. The particle mesh Ewald method calculated the long-range electrostatic interactions under periodic boundary conditions. The cut-off for the truncation of non-bonding interactions was 10 Å. SHAKE constrained all bonds involving hydrogen with an integration time step of 2 fs. The GPU version of PMEMD generated the trajectory through MD simulation^55^. A single run consisted of four stages: minimization, heating, equilibrium, and production. After 1500 cycles of minimization, the heat of the system was elevated from 0 K to 300 K at a constant volume for 50 ps. The equilibrium step consisted of a constant pressure (1 atm) and temperature (300 K) continued for the next 100 ps. Unrestrained equilibration for producing trajectories followed for 102 ns using a Langevin thermostat with a collision frequency of 2 ps^−1^ under a constant pressure of 1 atm. The first 2 ns were omitted and the analyses used the remaining 100 ns. A run was repeated three times with different random seeds. For MMPBSA analyses with **JJ1** and its analogs **JJ1a** and **JJ1b**, 20 ns MD simulations were employed for producing MD trajectories. Besides the duration, the other procedures were identical. “*MMPBSA.py*” was used to extract the change of free energy from the data^56^.

### Cell culture

Primary human umbilical vein endothelial cells (HUVECs) were obtained from Cambrex Bio Science (Charles City, IA, USA) and were maintained as previously described^57–59^. Briefly, cells were cultured to confluence in EBM-2 basal media supplemented with growth supplements (Cambrex Bio Science, Charles City, IA, USA) at 37 °C under an atmosphere of 5% CO_2_. All experiments were performed using HUVECs at passage 3–5.

### Animals and husbandry

Male C57BL/6 mice (6–7 weeks old, weighing 27 g) were purchased from Orient Bio Co. (Sungnam, Republic of Korea) and were used after a 12-day acclimatization period. Five mice were housed in each polycarbonate cage under controlled conditions: temperature (20–25 °C), relative humidity (40–45%), and a 12:12 h light:dark cycle. The mice received a normal rodent-pellet diet and water *ad libitum* during acclimatization and treatment. All animals were treated and all experiments were carried out in accordance with the guidelines and the regulations of the Guidelines for the Care and Use of Laboratory Animals approved and issued by Kyungpook National University, Republic of Korea (IRB No. KNU 2016-54). The protocols were approved by the Institutional Animal Care and Use Committee of the Kyungpook National University.

### Pharmacokinetic parameters

The penile veins of male C57BL/6 mice were injected with 2 mg/kg **JJ1**. Approximately 50 μL of blood was obtained from the tail vein immediately before and at 5, 15, 30, 60, and 180 min after injection; 80 μL acetonitrile and internal standard solution (reserpine) were then added to these samples, and plasma samples (10 μL) were prepared by centrifugation of the blood at 13,000 rpm for 15 min at 4 °C. An aliquot (2 μL) of the supernatant was then injected into an HPLC column. The pharmacokinetic parameters were determined using the standard non-compartmental method. The area under the curve for serum was calculated using the log linear trapezoidal method on WinNonlin (version 2.0; Scientific Consulting, KY, USA). The samples were analyzed using an Accela^TM^ LC system coupled to a TSQ Vantage triple quadrupole mass spectrometer (Thermo Fisher Scientific Inc., USA) equipped with a HESI-II Spray source. For the LC analysis, an ACE^®^ 5C18, 3 μm (2.1 × 50 mm) column was used. The mobile phases were (A) LC-grade^®^ water containing 0.1% formic acid, and (B) LC grade acetonitrile containing 0.1% formic acid (B). This initial composition was increased to 95% solvent (B) for 3 minutes. The gradient program for HPLC used a flow rate of 230 mL/min. Electrospray ionization was performed in positive mode at a spray voltage of 3,500 V. Nitrogen was used as both the sheath and auxiliary gas at optimum values of 45 arbitrary units and 20 arbitrary units, respectively. The vaporizer and capillary temperatures were 150 °C and 300 °C, respectively.

### Isolation of human plasma and platelets

Human blood samples were collected in the morning from 10 healthy, fasted volunteers (age: 24–28 years; four men, six women) without cardiovascular disorders, allergies, and lipid or carbohydrate metabolism disorders, and received drug treatment. All subjects gave written informed consent before participation in the study. The subjects did not use addictive substances or antioxidant food supplementation and consumed a balanced diet (meat and vegetables). Blood samples were collected in sodium citrate (0.32% final concentration, 10.9 mM), immediately centrifuged (1,300 *g* × 15 min) in order to obtain plasma, and pooled plasma was used for further study. Human platelets were prepared as previously described^60,61^. Briefly, platelet-rich plasma (PRP) was prepared by centrifugation at room temperature for 15 min at 150 *g*. PRP was adjusted to a concentration of 1 × 10^9^ platelets/mL with the use of a hemocytometer for cell counts. PRP was washed once with HEPES buffer (5 mM HEPES, 136 mM NaCl, 2.7 mM KCl, 0.42 mM NaH_2_PO_4_, 2 mM MgCl_2_, 5.6 mM glucose, 0.1% BSA (w/v); pH 7.45) in the presence of 1 mM CaCl_2_. The platelets were left at room temperature for 30 min. A sample volume of 10 mL was used for each clotting time point measurement. All participants and their legal guardians provided written informed consent and all experimental protocols were performed in accordance with the Ethics Committee on the Institutional Review Board of Kyungpook National University Hospitals (Study protocol No. KNUH 2012-01-010, Daegu, Republic of Korea). The protocols were approved by the Institutional Animal Care and Use Committee of the Kyungpook National University.

### *In vitro* and *ex vivo* platelet aggregation assay

The *in vitro* platelet aggregation study was performed according to a previously reported method^59,62^. Washed human platelets were incubated with the indicated concentration of **JJ1** in DMSO for 1, 3, 5, or 10 min and subsequently stimulated by thrombin in 0.9% saline solution at 37 °C for 5 min. Platelet aggregation was recorded using an aggregometer (Chronolog, Havertown, PA, USA). For the *ex vivo* aggregation assay, male mice were fasted overnight and the indicated concentration of **JJ1** in DMSO was administered by i.v. injection. After 24 h, PRP (10^9^ platelets/mL) in a total volume of 240 μL was incubated at 37 °C for 1.5 min in the aggregometer with continuous stirring at 1000 rpm and subsequently stimulated with thrombin. Platelet aggregation was recorded as described above.

### Cell viability assay

The MTT assay was used as an indicator of cell viability. Cells were grown in 96-well plates at a density of 5 × 10^3^ cells/well. After 24 h, the cells were washed with fresh medium and treated with **JJ1**. After a 48-h incubation period, the cells were washed and 100 μL of 1 mg/mL MTT was added to the cells and followed with a further 4-h incubation period. Finally, 150 μL DMSO was added to solubilize the formed formazan salt, and the amount of product was determined by the measurement of the absorbance at 540 nm using a microplate reader (Tecan Austria GmbH, Grödig, Austria).

### Anticoagulation assay

The aPTT and PT were determined using a Thrombotimer (Behnk Elektronik, Norderstedt, Germany) in accordance with the manufacturer’s instructions and as previously described^63^. Briefly, citrated normal human plasma (90 μL) was mixed with 10 μL **JJ1** and incubated for 1 min at 37 °C. Subsequently, the aPTT assay reagent (100 μL) was added, the plasma sample was incubated for an additional 1 min at 37 °C, 20 mM CaCl_2_ (100 μL) was added, and the clotting times were recorded. For the PT assays, citrated normal human plasma (90 μL) was mixed with 10 μL **JJ1** stock solution and was incubated for 1 min at 37 °C. The PT assay reagent (200 μL), which had been pre-incubated for 10 min at 37 °C, was subsequently added and the clotting time was recorded. The PT results were expressed in seconds and as International Normalized Ratios (INR): INR = (PT sample/PT control)^ISI^, where ISI = international sensitivity index. The aPTT results were also expressed in seconds. All experimental protocols (KNUH 2012-01-010) were approved by the Institutional Review Board of Kyungpook National University Hospitals (Daegu, Republic of Korea).

### *In vivo* bleeding time

Tail bleeding times were measured by using the method of Dejana *et al*.^63,64^. Briefly, C57BL/6 mice were fasted overnight prior to the experiments. One hour after the i.v. administration of **JJ1**, the tails of the mice were transected at 2 mm from their tips. The bleeding time was defined as the time elapsed until the bleeding stopped. Bleeding times exceeding 15 min were recorded as lasting for 15 min.

### *Ex vivo* clotting time

Male C57BL/6 mice were fasted overnight. Each compound was dissolved in 0.5% DMSO and administered by i.v. injection. One hour after administration, arterial blood samples (0.1 mL) were collected in 3.8% sodium citrate (1:10, v/v) for the *ex vivo* aPTT and PT determination. The clotting times were determined as described above.

### Determination of *in vitro* enzyme inhibition

The inhibitor constants (*K*_*i*_) were determined for the inhibition of a series of human enzymes by **JJ1**, efegatran, and argatroban. Chromogenic substrate assays were performed using a Labsystems IEMS (Cergy Pontoise, France) microtiter plate reader. *K*_*i*_ values were calculated according to the method of Dixon^65^. In each assay, the compound was tested at a minimum of seven concentrations, in duplicate, to obtain an inhibition curve. The following general procedures were adopted for each assay. In a 96-well microtiter plate, 25 μL of inhibitor solution or buffer was added to 50 μL of substrate. The enzyme solution (25 μL) was added just before the plate was placed in the microtiter plate reader for 1 h at 37 °C. The hydrolysis of the substrate yields *p*-nitroaniline, which was continuously monitored spectrophotometrically at 405 nm. The maximal initial reaction rates were calculated and expressed as millioptical density per minute. Curve fitting (Dixon plot of 1/V_max_ versus inhibitor concentration) was performed by linear regression analysis to calculate the *K*_*i*_ value.

### Thrombin-catalyzed fibrin polymerization

Thrombin-catalyzed polymerization was determined every 6 s for 20 min by monitoring the turbidity of the solution at 360 nm at ambient temperature by using a spectrophotometer (TECAN, Männedorf, Switzerland). Control plasma and plasma incubated with **JJ1** were diluted three-fold in DMSO and clotted with thrombin (final concentration: 0.5 U/mL). The maximum polymerization rate (Vmax, ΔmOD/min) from each absorbance curve was recorded^66^.

### ELISA for plasma fibrinopeptide A (FPA)

The generation of FPA in plasma with or without **JJ1** was quantified in accordance with the manufacturer’s instructions by using a commercially available ELISA kit (LSBio, Seattle, WA). Values were measured using an ELISA plate reader (Tecan, Austria GmbH, Austria).

### Arterial thrombosis animal model

The FeCl_3_-induced thrombosis mouse model was established as previously described^27^. Male C57BL/6 mice were fasted overnight and were administered **JJ1** in DMSO by intravenous injection. Then, mice were anesthetized using 3% isoflurane (Forane®, Choongwae Pharma. Corp., Seoul, Korea) and injected intravenously with 0.1 mL 0.1% rhodamine 6 G (Sigma). A testicular artery (200 μm in diameter) was carefully exposed and a cotton thread (200 μm in diameter) saturated with 0.25 mol/L FeCl_3_ was applied to the adventitial surface. After 5 min, the cotton thread was removed, and the wound was flushed with saline solution. Thrombus formation was monitored at 35 °C by 3-dimensional imaging as previously described^67^. The size and time of thrombus formation were monitored, and the findings were scored as follows: no thrombus, 0; small thrombus (50 μm × 75 μm), 1; medium-sized thrombus (100 μm × 150 μm), 2; large thrombus (200 μm × 300 μm), 3. The time from FeCl_3_-mediated endothelial injury to occlusion of the testicular artery by a large thrombus was measured.

### Acute thrombosis induced by a combination of collagen and epinephrine in mice

Male C57BL/6 mice were fasted overnight and divided into groups of 10 animals. **JJ1** suspended in DMSO was administered to mice intravenously. After 1 h, a mixture of collagen (500 μg/kg) plus epinephrine (50 μg/kg) was injected into the tail vein of mice to induce acute thrombosis 1 h later. Each mouse was carefully examined for 15 min to determine whether the mouse was paralyzed, dead, or had recovered from the acute thrombotic challenge. Five separate experiments were performed and the results were subjected to statistical analysis.

### Production of thrombin on the surfaces of HUVECs

Thrombin production by HUVECs was quantified as previously described^63,68^. Briefly, HUVECs were pre-incubated in 300 μL of a solution containing **JJ1** in 50 mM Tris-HCl buffer, 100 pM FVa, and 1 nM FXa for 10 min, followed by the addition of prothrombin to a final concentration of 1 μM. After 10 min, duplicate samples (10 μL each) were transferred into a 96-well plate containing 40 μL 0.5 M EDTA in TBS per well to terminate the prothrombin activation. Activated prothrombin was determined by the measurement of the rate of hydrolysis of S-2238 (a thrombin substrate) at 405 nm. Standard curves were prepared with known amounts of purified thrombin.

### Thrombin activity assay

**JJ1** was mixed into 50 mM Tris-HCl buffer (pH 7.4) containing 7.5 mM EDTA and 150 mM NaCl. Following a 2-min incubation at 37 °C, thrombin solution (150 μL, 10 U/mL) was added, followed by incubation at 37 °C for 1 min. A solution of the thrombin substrate, S-2238, (150 μL, 1.5 mM) solution was subsequently added and the absorbance at 405 nm was monitored for 120 s using a spectrophotometer (TECAN, Männedorf, Switzerland).

### Measurements of organ injury markers

Serum levels of AST, ALT, BUN, and creatinine were measured using commercial assay kits (Pointe Scientific, Linclon Park, MI).

### Statistical analysis

The results are expressed as the mean ± SD of at least three independent experiments performed in duplicate. *P* < 0.05 was considered statistically significant and was determined using the SPSS software (version 14.0, SPSS Science, Chicago, IL, USA). Statistical relevance was determined by one-way analysis of variance (ANOVA) and Tukey’s post-hoc test.

## Electronic supplementary material

Supplementary Dataset
