# Supplementary material for: Antithrombotic properties of JJ1, a potent and novel thrombin inhibitor
Source: Sci Rep. 2017 Nov 1;7:14862. doi: 10.1038/s41598-017-13868-1 (PMC5665883; doi:10.1038/s41598-017-13868-1)
Supplement: Supplementary file 1 — Supplementary Dataset [file 41598_2017_13868_MOESM1_ESM.doc]

# Antithrombotic properties of JJ1, a potent and novel thrombin inhibitor

# Wonhwa Lee^1^, Suyeon Lee^1^, Joonhyeok Choi^1^, Jun-Hyeong Park^1^, Kyung-Min Kim^2^, Jun-Goo Jee^1^*, and Jong-Sup Bae^1^*

From the ^1^College of Pharmacy, CMRI, Research Institute of Pharmaceutical Sciences, BK21 Plus KNU Multi-Omics based Creative Drug Research Team, Kyungpook National University, Daegu 41566 Republic of Korea; ^2^Division of Plant Biosciences, School of Applied BioSciences, College of Agriculture and Life Science, Kyungpook National University, Daegu 41566 Republic of Korea

Running title: **JJ1**, a new thrombin inhibitor

# * Corresponding Authors:

# Jun-Goo Jee, Ph.D.

College of Pharmacy, Kyungpook National University

# 80 Daehak-ro, Buk-gu, Daegu 41566, Republic of Korea

# Phone, 82-53-950-8568; Fax, 82-53-950-8557

# Email: jjee@knu.ac.kr

# and

# Jong-Sup Bae, Ph.D.

College of Pharmacy, Kyungpook National University

# 80 Daehak-ro, Buk-gu, Daegu 41566, Republic of Korea

# Phone, 82-53-950-8570; Fax, 82-53-950-8557

# Email: baejs@knu.ac.kr

**Table S1.** **Information regarding 40 molecules purchased for analysis**

| No. | ZINC ID | Score (kcal/mol) | Rank | Max. Tc | Closest known inhibitor |
| --- | --- | --- | --- | --- | --- |
| 1 | ZINC10450189 | -87.13 | 2 | 0.272 | ZINC29125652 |
| 2 | ZINC37693613 | -85.41 | 3 | 0.213 | ZINC13808035 |
| 3 | ZINC04991109 | -69.21 | 5 | 0.3 | ZINC13808146 |
| 4 | ZINC09063750 | -68.36 | 7 | 0.239 | ZINC03807246 |
| 5 | ZINC02885063 | -67.5 | 8 | 0.271 | ZINC01646573 |
| 6 | ZINC01115725 | -66.76 | 9 | 0.377 | ZINC06405865 |
| 7 | ZINC12002652 | -66.34 | 11 | 0.206 | ZINC00037095 |
| 8 | ZINC09668789 | -65.19 | 12 | 0.269 | ZINC00115253 |
| 9 | ZINC02882350 | -60.48 | 17 | 0.286 | ZINC06405865 |
| 10 | ZINC01056557 | -60.14 | 18 | 0.208 | ZINC05751469 |
| 11 | ZINC06697730 | -59.46 | 19 | 0.212 | ZINC13808118 |
| 12 | ZINC01834017 | -59.26 | 22 | 0.298 | ZINC06405865 |
| 13 | ZINC04990100 | -58.69 | 24 | 0.222 | ZINC14210470 |
| 14 | ZINC01078720 | -58.67 | 25 | 0.375 | ZINC06405865 |
| 15 | ZINC00190819 | -58.36 | 26 | 0.169 | ZINC00037095 |
| 16 | ZINC15017614 | -58.09 | 27 | 0.213 | ZINC04426028 |
| 17 | ZINC04755823 | -58.03 | 28 | 0.205 | ZINC00115253 |
| 18 | ZINC13496962 | -57.84 | 31 | 0.263 | ZINC01665651 |
| 19 | ZINC08659817 | -57.62 | 34 | 0.26 | ZINC01487441 |
| 20 | ZINC02443067 | -57.58 | 36 | 0.143 | ZINC02006270 |
| 21 | ZINC09313441 | -57.14 | 39 | 0.218 | ZINC02006270 |
| 22 | ZINC67629459 | -57.14 | 40 | 0.259 | ZINC28238972 |
| 23 | ZINC13637778 | -57.05 | 44 | 0.152 | ZINC13808067 |
| 24 | ZINC05023167 | -56.87 | 45 | 0.186 | ZINC00037188 |
| 25 | ZINC44894084 | -56.62 | 47 | 0.207 | ZINC11677837 |
| 26 | ZINC41146472 | -56.35 | 53 | 0.273 | ZINC01487441 |
| 27 | ZINC08310933 | -56.32 | 55 | 0.238 | ZINC01490578 |
| 28 | ZINC09289128 | -56.19 | 56 | 0.206 | ZINC14113515 |
| 29 (*) | ZINC41152207 | -56.01 | 59 | 0.207 | ZINC00410207 |
| 30 | ZINC19357055 | -55.96 | 61 | 0.236 | ZINC03814057 |
| 31 | ZINC00290408 | -55.82 | 62 | 0.213 | ZINC04426028 |
| 32 | ZINC18115797 | -55.73 | 66 | 0.206 | ZINC13808067 |
| 33 | ZINC40267470 | -55.66 | 68 | 0.174 | ZINC03807246 |
| 34 | ZINC06292411 | -55.43 | 73 | 0.271 | ZINC01646573 |
| 35 | ZINC06726175 | -55.19 | 81 | 0.205 | ZINC04426028 |
| 36 | ZINC41151081 | -54.97 | 89 | 0.259 | ZINC26665352 |
| 37 | ZINC13497423 | -54.85 | 92 | 0.291 | ZINC06864978 |
| 38 | ZINC00055120 | -54.8 | 96 | 0.306 | ZINC00036634 |
| 39 | ZINC15837847 | -54.75 | 99 | 0.264 | ZINC01705403 |
| 40 | ZINC04985999 | -54.37 | 119 | 0.311 | ZINC03276773 |

**Figure S1.** **Distribution and correlation of AUC and LogAUC**


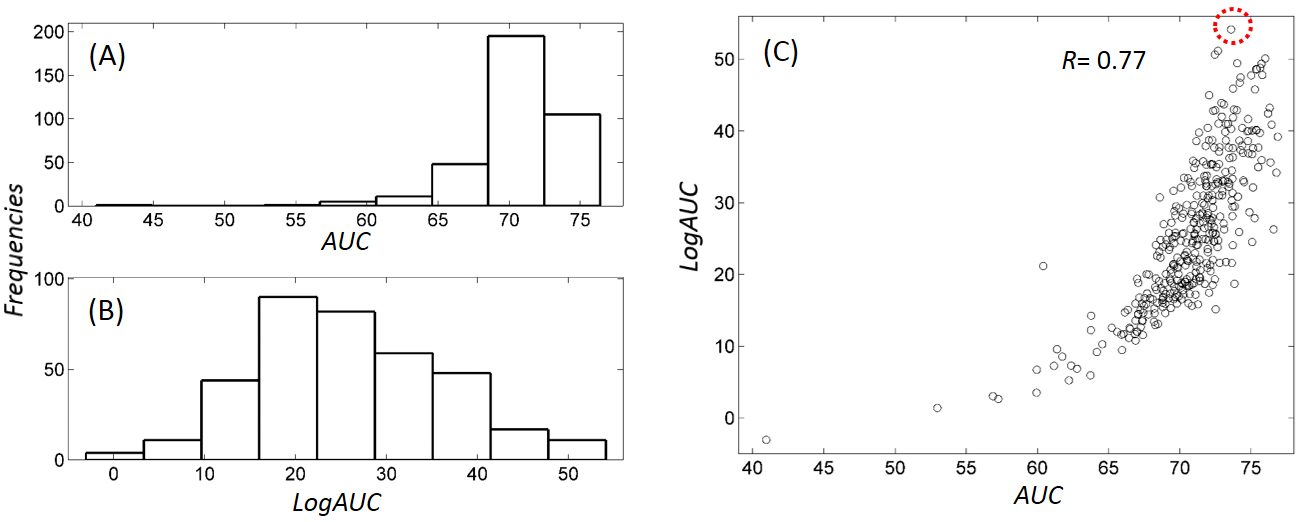


**Figure S2.** **Chemical structures of 40 molecules purchased for analysis**


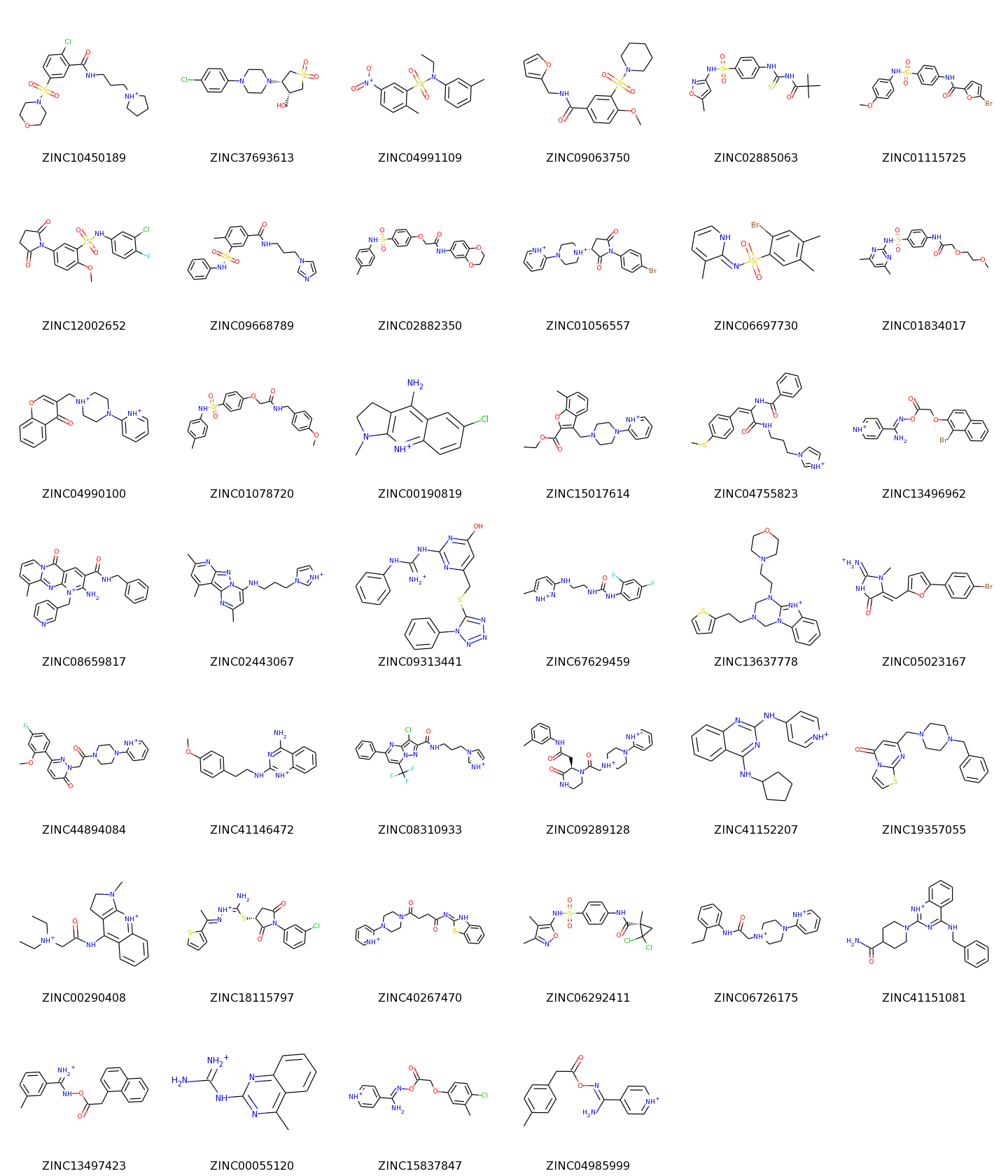


**Figure S3.** **Input file for docking with DOCK 3.6**

DOCK 3.5 parameter

# ----- INPUT -----

receptor_sphere_file rec_sph/match2.sph

ligand_atom_file ZINC41152207.ddb

output_file_prefix test.

cluster_numbers 1

random_seed 100133

# ----- MATCHING -----

distance_tolerance 1.2

nodes_maximum 4

nodes_minimum 4

ligand_binsize 0.2

ligand_overlap 0.1

receptor_binsize 0.2

receptor_overlap 0.1

bump_maximum 1

focus_cycles 0

focus_bump 0

focus_type energy

critical_clusters no

# ----- COLORING -----

chemical_matching yes

case_sensitive no

match positive negative

match positive negative_or_acceptor

match positive not_neutral

match negative positive

match negative positive_or_donor

match negative not_neutral

match donor acceptor

match donor donacc

match donor negative_or_acceptor

match donor neutral_or_acceptor_or_donor

match donor not_neutral

match acceptor donor

match acceptor donacc

match acceptor positive_or_donor

match acceptor neutral_or_acceptor_or_donor

match acceptor not_neutral

match neutral neutral

match neutral neutral_or_acceptor_or_donor

match ester_o donor

match ester_o donacc

match ester_o positive_or_donor

match ester_o not_neutral

match amide_o donor

match amide_o donacc

match amide_o positive_or_donor

match amide_o not_neutral

# ----- SEARCH MODE -----

ligand_desolvation volume

ratio_minimum 0.0

atom_minimum 5

atom_maximum 60

number_save 500

molecules_maximum 300000

restart_interval 10000

initial_skip 0

timeout 20

# ----- SCORING -----

solvmap_file rec_grids/solvmap.heavy

#hydrogen_solvmap_file rec_grids/solvmap.hydro

delphi_file rec_grids/rec+sph.phi

chemgrid_file_prefix rec_grids/chem

vdw_parameter_file rec_grids/vdw.parms.amb.mindock

check_clashes yes

remove_positive_solvation no

vdw_maximum 1.0e10

electrostatic_scale 1.0

vdw_scale 1.0

delphi_nsize 179

# ----- MINIMIZATION -----

minimize yes

minimization_max 1.0e15

check_degeneracy no

simplex_iterations 1000

simplex_convergence 0.1

simplex_restart 1.0

simplex_initial_translation 0.2

simplex_initial_rotation 5.0

**Figure S4.** **Binding free energies of JJ1 and analogs with MMPBSA**


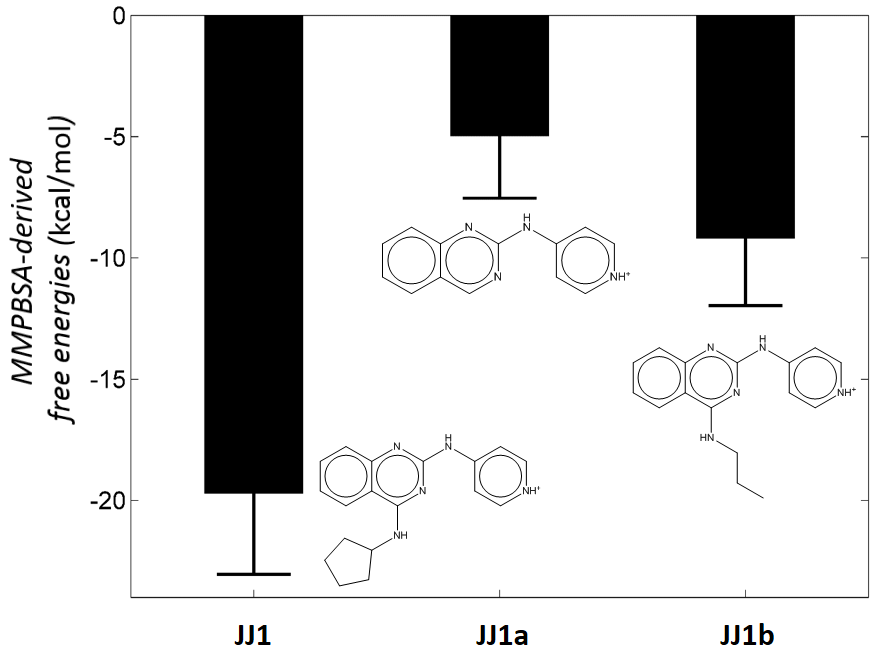


**Table S1.** **Information regarding 40 molecules purchased for analysis.** The rankings and energies of the 40 molecules selected by high throughput virtual screening for the enzyme assay are listed. “Rank” is the order by the scoring function of DOCK 3.6 and “Max Tc” means the maximum value of Tanimoto coefficient with the Morgan circular fingerprint between the molecule and all BindingDB-registered thrombin inhibitors. The molecule (*) indicates JJ1 of the current study.

**Figure S1.** **Distribution and correlation of AUC and LogAUC.** The thrombin inhibitors (true positives) extracted from BindingDB and their physicochemically matched but topologically different decoys (false positives) were docked into the 366 thrombin crystal structures. The enrichments of the true positives over the false positives were calculated using the area under the curve (AUC) in the receiver operating characteristics (ROC) curves. (A) Histogram of the distribution of AUC values. (B) Histogram of the distribution of LogAUC values. (C) Correlation between AUC and LogAUC. The case of 2CF9-H is marked as a dotted circle.

**Figure S2. Chemical structures of 40 molecules purchased for analysis.** The 40 molecules purchased for high throughput virtual screening for the enzyme assay are drawn according to their rankings. The corresponding ZINC IDs are labeled.

**Figure S3.** **Input file for docking with DOCK 3.6.** The content of the INDOCK file for controlling docking in each molecule is shown. The term for “ZINC41152207.ddb” is replaced for each molecule. The flexibase format file, ZINC41152207.ddb, was downloaded from the ZINC12 website (<http://zinc.docking.org/substance/ZINC41152207>).

**Figure S4.** **MMPBSA analyses of JJ1 and analogs.** The mean (± standard deviation) values of MMPBSA-derived free energy for **JJ1**, **JJ1a**, and **JJ1b** were extracted in three independent runs with different random seeds as -19.7 (± 3.3), -5.0 (± 2.6), and -9.2 (± 2.8) (kcal/mol), respectively.
